# Clinical features of anti-neutrophil cytoplasmic antibody-associated systemic vasculitis in French children

**DOI:** 10.1186/1546-0096-9-S1-P88

**Published:** 2011-09-14

**Authors:** AS Sacri, B Ranchin, B Florkin, H See, T Ulinski, H Flodrops, S Decramer, R Salomon, P Quartier, J Harambat

**Affiliations:** 1 Department of Pediatric Nephrology, CHU Pellegrin, Bordeaux, France; 2 Department of Pediatric Nephrology, HFME, Lyon, France; 3 Department of Pediatric Rhumatolgy, Necker Enfants Malades, Paris, France; 4 Department of Pediatric Nephrology, CHU Robert Debré, Paris, France; 5 Department of Pediatric Nephrology, CHU Armand Trousseau, Paris, France; 6 Department of Pediatric Nephrology, CHR Réunion, France; 7 Department of Pediatric Nephrology, CHU Toulouse, France; 8 Department of Pediatric Nephrology, Necker Enfants Malades, Paris, France

## Background

Anti-neutrophil cytoplasmic antibody (ANCA)-associated vasculitis is mainly reported in adults. Data in children is scarce. The current study aimed to describe the clinical features of the disease at diagnosis and the long-term outcomes in pediatric patients with ANCA-associated vasculitis.

## Methods

This retrospective study was conducted in 9 French Hospitals (mainly pediatric nephrology units) and includes patients diagnosed during the past 25 years. Wegener’s granulomatosis (WG) and microscopic polyangiitis (MPA) were included, but not Churg-Strauss syndrome.

## Results

Forty-seven children were included, 15 (32%) with WG and 32 (68%) with MPA, 77% children were pANCA+/MPO. Female predominated (85%), the median age at diagnosis was 10 years (2-16), and average time to diagnosis was 8 months (0-58). 53% children presented with fever, 77% with deterioration of general condition. 91% had renal involvement. Among them 91% had proteinuria, 44% had hypertension, 79% had renal failure of whom one third presented with end-stage renal disease (ESRD), 22% had gross hematuria and 82% microscopic hematuria. Half the patients showed pulmonary involvement (alveolar hemorrhage for 77% and nodules for 18%). 28% presented with upper airways involvement, 38% with cutaneous lesions (67% purpura), and 19% with arthritis. Few had digestive, ophthalmic or CNS involvement. After an average follow-up of 5 years (2m-24y), 3 patients (6%) died. 60% were considered in remission (partial or total) after induction treatment and 77% at last follow-up. 33% of children with initial renal involvement developed chronic kidney disease, half of them progressed to ESRD. 43% needed dialysis and 29% underwent a renal transplantation.

## Conclusion

Clinical features and outcome in children with ANCA-vasculitis are close to the findings in adults but they are characterized by delayed diagnosis and female predominance. Although the cases may be selected, renal involvement appears as a major prognostic factor of the disease.

